# Multi-Organ Transcriptomic Analysis of Greater Amberjack (*Seriola dumerili*) with Different Growth Rates

**DOI:** 10.3390/ani16030516

**Published:** 2026-02-06

**Authors:** Xiaoying Ru, Xiaojing Li, Yang Huang, Peipei Chen, Qiuxia Deng, Hang Li, Qibing Zhou, Haoyi Lin, Ruijuan Hao, Yongguan Liao, Jinhui Wu, Yanfei Zhao, Chunhua Zhu

**Affiliations:** 1 Guangdong Research Center on Reproductive Control and Breeding Technology of Indigenous Valuable Fish Species, Guangdong Provincial Key Laboratory of Aquatic Animal Disease Control and Healthy Culture, Fisheries College of Guangdong Ocean University, Zhanjiang 524088, China; 2 Development and Research Center for Biological Marine Resources, Southern Marine Science and Engineering Guangdong Laboratory (Zhanjiang), Zhanjiang 524025, China; 3 China National Fisheries Corporation, Beijing 100160, China; 4 Agro-Tech Extension Center of Guangdong Province, Guangzhou 510500, China

**Keywords:** *Seriola dumerili*, hypothalamus-pituitary-liver, growth rates, transcriptomic analysis

## Abstract

In order to explore the main regulatory genes and related pathways of growth traits, transcriptome sequencing was performed on the hypothalamus, pituitary, and liver tissues of 12-month-old greater amberjack (*Seriola dumerili*) with different growth rates. In total, 504 (118 up- and 386 down-regulated), 556 (283 up- and 273 down-regulated), and 699 (224 up- and 475 down-regulated) differentially expressed genes (DEGs) were identified in the hypothalamus, pituitary, and liver tissues, respectively. GO and KEGG pathway analyses revealed significant differences in the expression of several genes involved in growth, metabolism, and immune-related pathways. The mRNA expression levels of genes related to growth (*ghrh*, *ghra*, *igf1*), cell proliferation (*fgf19*, *fgfr4*, *mapk8b*, *map2k4b*, and *map4k3*), and lipid metabolism (*acsl5*, *dgat2*, *lipeb*, *cyp7a1*, and *fabp10a*) were up-regulated in the fast-growing (FG) group, while the *cartl* and *sst1.1* were down-regulated. Conversely, genes associated with glycolysis (*fbp1a*, *pklr*, *pgm2*), citrate cycle (*aclya*, *idh1*), and immune-related pathways (*irf1b*, *cxcl10*, *tnfb*, *lysg*, *ifi44*, *mapk11*, and *mapk12b*) were up-regulated in the slow-growing (SG) group. These findings indicate that the FG exhibited greater lipid metabolism capacity and cell proliferation ability, while the SG expended additional energy to cope with environmental stress, with hindered growth during immune response. This study enhances our understanding of the genetic mechanisms underlying differences in growth rates and provides essential gene resources for future growth-related molecular breeding programs in greater amberjack.

## Simple Summary

The greater amberjack (*Seriola dumerili*) is a highly valued fish known for its tasty meat, nutritional richness, and rapid growth rate. However, the molecular mechanisms underlying its growth traits remain poorly understood. In this study, transcriptome sequencing was performed on the hypothalamus, pituitary, and liver tissues of 12-month-old greater amberjack with different growth rates. A number of genes and biological pathways that related to growth, cell proliferation, lipid and glucose metabolism, and immune responses were screened. These findings contributes to a deeper understanding of the growth control mechanism in greater amberjack and offers a scientific basis for efficient breeding and species improvement.

## 1. Introduction

Growth is one of the most important economic traits in animal breeding. In aquaculture, variability among individuals significantly hampers productivity and profitability. Therefore, analyzing the potential mechanisms of growth differences among individuals provides critical insights to inform the design of more efficient breeding strategies [1]. The differentiation in the growth and development of animals is influenced by the interplay of various environmental factors, including season, temperature, nutritional manipulation, and the endogenous genetic background [1,2]. Genetic background is a critical consideration when breeding new strains with faster growth rates. Similarly to other vertebrates, both the brain and liver participate in the hypothalamus–pituitary–liver growth axis, which is one of the most crucial pathways in the regulation of fish growth [3]. In this axis, the hypothalamus regulates the release of growth hormone from the pituitary gland, which in turn modulates liver function, including the release and metabolism of insulin-like growth factor. The major genes involved in this axis include growth hormone releasing hormone (*ghrh*) and somatostatin (*ss*) secreted by the hypothalamus, growth hormone (*gh*) secreted by the pituitary gland, and insulin-like growth factor-I (*igf1*) released by target organs such as the liver [2,4]. Although many studies have demonstrated the functions of these genes in the growth axis, the comprehensive regulatory network connecting these genes to their direct phenotypes and growth rates in various animals remains largely unexplored.

The elucidation of the molecular mechanisms, genes, and signaling pathways involved in growth has significant implications for various growth-related research areas. With the advancement of high-throughput sequencing, numerous studies have concentrated on exploring candidate genes and regulatory pathways associated with fish growth through RNA-seq. Lv et al. employed RNA-seq to analyze the muscle, liver, and brain tissues of rock carp (*Procypris rabaudi*). Gene Ontology (GO) and Kyoto Encyclopedia of Genes and Genomes (KEGG) enrichment analyses revealed that differentially expressed genes (DEGs) were predominantly involved in the regulation of appetite, lipid transport and metabolism, protein degradation and digestion, and glycolysis/gluconeogenesis [5]. Zhang et al. conducted transcriptome analyses of liver and muscle tissues from black carp (*Mylopharyngodon piceus*) of varying body sizes and identified several genes involved in growth- and development-related metabolic pathways, including the FoxO, p53, PI3K-Akt, apoptosis, TGF-β, and insulin signaling pathways [6]. Li et al. analyzed the transcriptomes of the muscles of three-month-old rice flower carp (*Cyprinus carpio*) with different body sizes and discovered several genes associated with the ubiquitin–proteasome pathway and muscle contraction [7]. This method has also been used in other aquatic organism with similar objectives, such as Qingbo (*Spinibarbus sinensis*) [8], large yellow croaker (*Larimichthys crocea*) [9], blunt-snout bream (*Megalobrama amblycephala*) [10], Pacific black-lipped pearl oyster (*Pinctada margaritifera*) [11], channel catfish (*Ictalurus punctatus*) [12], and Chinese longsnout catfish (*Leiocassis longirostris*) [13], etc. Our findings indicate a lack of comparative transcriptomic analyses of endocrine and immune regulation, as well as metabolic patterns, in the hypothalamic–pituitary–liver axis among individuals with growth differences in greater amberjack (*Seriola dumerili*).

The greater amberjack is an important marine commercial fish species found along the coasts of Hainan Island, Guangdong, Fujian, and Zhejiang in China. Due to its rapid growth, large body size, and high-quality flesh, the greater amberjack exhibits high commercial value and is one of the key aquatic products for export in China [14]. Wild female greater amberjack reach maturity at 1–5 years of age, with a mean age of 1.3 years for 50% of females to mature, indicating their rapid growth, early maturation, and high fecundity [15,16]. However, it has been reported that the growth of the greater amberjack is restricted in individuals reared in captivity. Slow-growing fish present a bottleneck in aquaculture due to the high mortality rates of smaller individuals, which can lead to inefficiencies in aquaculture if they constitute a significant proportion of the stock [17]. Therefore, it is essential to conduct relevant research on the growth characteristics of greater amberjack. Accelerating their growth rate could shorten the culture cycle, reduce farming costs, increase production and provide economic benefits by meeting the high market demand for this species. Nevertheless, current research on greater amberjack primarily focuses on reproductive biology [18,19], stress physiology [20,21], feed nutrition [22], and disease resistance and immunity [23]. There have been few studies on the genetic mechanisms underlying its growth. Furthermore, the foundational research for large-scale selective breeding and trait improvement remains relatively weak. In this study, we collected greater amberjack of two different sizes, obtained hypothalamus, pituitary, and liver tissues, and investigated their growth characteristics at the transcriptome level using RNA-seq. We employed the Illumina NovaSeq 6000 platform to acquire high-quality data, expression profiles, and DEGs from eighteen libraries. The findings of this study will provide foundational information for enhancing the growth traits of greater amberjack and contribute to further functional studies of growth-related genes.

## 2. Materials and Methods

### 2.1. Animals and Tissue Sampling

The same batch of artificially bred, 12-month-old F1-generation greater amberjack selected for this study was derived from a breeding population with multiple unrelated parental pairs and originated from the Leizhou Island (Zhanjiang, China). The dissolved oxygen level exceeded 6 mg/L, the salinity was measured at 30.4  ±  0.5‰, the annual variation in water temperature ranged from 19.1 to 30.3 °C, and the pH was 8.4  ±  0.1. The fish were cultured in offshore cages (5 m × 5 m × 4 m), with 400 juvenile fish per cage. They were fed commercial feed twice daily (at 9:00 a.m. and 6:00 p.m.) at 3% of their body weight. After 12 months of rearing, two groups were formed based on phenotype by selecting greater amberjack with different growth rates within the same population of 12 month olds, resulting in fast- and slow-growing groups. Subsequently, 10 fish were randomly selected from each group for precise measurement. The final sample was determined by excluding abnormal individuals and applying strict weight thresholds (FG: 1000–1300 g, SG: 600–800 g). The average body weight, total length, and body height were recorded as 1149.16 ± 28.13 g, 43.74 ± 30.52 cm, and 10.7 ± 0.14 cm for the fast-growth group (FG: *n* = 7) and 686.76 ± 0.14 g, 37.32 ± 0.63 cm, and 9.11 ± 0.15 cm for the slow-growing group (SG: *n* = 6), respectively. The phenotype data for each individual are shown in Table 1. Prior to sample collection, the experimental fish were anesthetized using tricaine methane sulfonate (MS-222, Sigma, Saint Louis, MO, USA) at a concentration of 100 mg/L. The hypothalamus, pituitary, and liver tissues were rapidly isolated and preserved in RNAlater^®^ (Beyotime, Shanghai, China) overnight, and then frozen at −80 °C for RNA extraction. To eliminate the influence of sex on growth rates, three female greater amberjack from each group were selected for transcriptome sequencing. The sex of these samples was determined by dissecting the gonads. The hypothalamus, pituitary, and liver of fast-growing fish are designated as FH, FP, and FL, respectively, while those of slow-growing fish are designated as SH, SP, and SL, respectively.

### 2.2. RNA Extraction, Library Construction, and Sequencing

Total RNA was extracted from hypothalamic, pituitary, and liver tissues of greater amberjack using a Trizol kit (Invitrogen, Carlsbad, CA, USA) according to the manufacturer’s instructions. After contaminating genomic DNA was removed using DNase I (Takara Biotechnology, Tianjin, China), tRNA purity and concentration were measured using a Qubit 2.0 Fluorometer and a NanoDrop 2000 Spectrophotometer (Thermo Fisher Scientific, Waltham, MA, USA), while the RNA integrity was assessed using an Agient 2100 (Agilent Technologies, Palo Alto, CA, USA). The RNA integrity number (RIN) scores for the hypothalamus, pituitary, and liver tissues ranged from 6.2 to 8.6, indicating good overall RNA integrity and meeting the requirements for sequencing. Subsequently, high-quality mRNA was utilized for library construction. The procedures are described as follows: the bio-mRNA of the greater amberjack was enriched using Oligo (dT)-attached magnetic beads and fragmented in fragmentation buffer, with the disrupted mRNA serving as the template. A strand of cDNA was synthesized using a six-base random primer, followed by the addition of buffer solution, dNTP, RNase H, and DNA polymerase I to synthesize the second cDNA strand. The double-stranded cDNA was purified using AMPure Pure Beads and underwent end repair, had a poly(A)-tail added, and was connected to the sequencing adapter, followed by fragment size selection using AMPure XP Beads. Finally, the cDNA library was enriched by PCR. The quantity and quality of the constructed library were examined using the Agilent Bioanalyzer 2100 and Qubit 2.0, respectively. The libraries were sequenced on the Illumina NovaSeq6000 platform (Illumina, San Diego, CA, USA) at Biomarker Technologies Co., Ltd. (Beijing, China), generating 150 bp paired-end data. Three replicates (using one fish as a biological replicate) were obtained for transcriptome sequencing of FH, SH, FP, SP, FL, and SL.

### 2.3. Data Processing, Assembly, and Functional Annotation

First, the raw data quality was assessed using fastq (version 0.22.0). Adaptor sequences were removed and read pairs were quality filtered using in-house Perl scripts. The data quality control procedures included the following steps: (1) detachment of adaptor, (2) removal of low-quality reads containing more than 10% unknown nucleotides (N) and bases with a quality sore (Q) ≤ 10% (over 50% of the total read length) from the raw data. Concurrently, the Q20, Q30, and GC content of the clean data were calculated. The clean RNA-seq data were subsequently aligned to the greater amberjack reference genome (deposited in the DNA Data Bank of Japan (DDBJ) under accession numbers: BDQW01000001-BDQW01034655 (Biosample ID: SAMD00083043_sdu_WGS.acclist.zip) using HISAT2 (version 2.2.1). StringTie (version v2.2.1) was utilized to assemble the clean reads de novo. The N50 and N75 lengths were 3329 bp and 2156 bp, respectively.

The functions of the genes were determined by annotating them using several public databases, including the non-redundant protein (NR), Pfam (protein family), Swiss Prot (protein), KEGG, and GO annotation public databases. They were identified through NCBI BLASTx (version 2.2.26; https://blast.ncbi.nlm.nih.gov/, accessed on 27 July 2024) with an E-value threshold of 1 × 10^−5^ for NR (https://www.ncbi.nlm.nih.gov/refseq/, accessed on 27 July 2024), Swiss-Prot (http://www.uniprot.org/, accessed on 27 July 2024), and KEGG (http://www.genome.jp/kegg/, accessed on 27 July 2024) annotations. Additionally, the Pfam database was scanned using hmmscan (version 3.3.2) with an E-value threshold of 1 × 10^−5^. The Blast results were further analyzed with Blast2Go (version 2.5.0; http://www.blast2go.com/, accessed on 27 July 2024) to assign gene ontology to the mapped transcripts.

### 2.4. Differential Expression Analysis

Following the filtering steps, the raw counts of the remaining genes were normalized using the median of ratios method available in the R package DESeq2 (version 1.30.1). This normalization step corrects for differences in library size and RNA composition across samples. Subsequently, the normalized data were analyzed using DESeq2’s negative binomial model for accurate differential expression detection, which is designed to account for the discrete and over-dispersed nature of sequencing count data. Gene expression levels were quantified via the fragments per kb per million reads (FPKM) method. Principal component analysis (PCA) was conducted on the FPKM scores to evaluate the differences both between and within groups. Differential gene expression was assessed using the Wald test, with thresholds set at “*p*-value < 0.05” and “|log_2_Fold-change| > 1” to detect significant differences in transcriptional expression. DEGs exhibiting higher or lower expression levels than the slow-growth group in each tissue were classified as “up-regulated” or “down-regulated” genes, respectively. GO and KEGG enrichment analyses of the DEGs were then performed using the clusterProfiler (version 4.4.4) software based on the Wallenius noncentral hypergeometric distribution, which can be used to adjust for gene length bias in the DEGs. The pathways with a *p*-value < 0.05 were considered significant.

### 2.5. Validation of the RNA-Seq Analysis by RT-qPCR

The expression levels of growth-related genes in the hypothalamus, pituitary, and liver of the greater amberjack were detected using qPCR (with 5 biological replicates per group). Additionally, to further validate the reliability of the RNA-seq results, a total of 33 DEGs (14 up-regulated and 19 down-regulated DEGs, respectively) were randomly selected for qRT-PCR validation. This analysis included 3 biological replicates and 2 technical replicates. The samples used for qPCR validation experiments and RNA-seq were from the same batch. The primer sequences for these genes are provided in Table S1. Total RNA was extracted from the FH, SH, FP, SP, FL, and SL samples, and cDNA synthesis was performed using the PrimeScript™ RT Reagent Kit with gDNA Eraser (Takara Biotechnology, Shanghai, China), following the manufacturer’s instructions. qRT-PCR was conducted using a Light Cycler TM96 instrument (Roche, Indianapolis, IN, USA) with the PerfectStart^®^ Green qPCR SuperMix Kit (TransGen Biotech, Beijing, China). A 20 μL reaction mixture was prepared, consisting of 10 μL of 2 × SYBR Green Master Mix, 2 µL of template cDNA, 0.4 μL of each forward and reverse primer 10 μM, and 7.2 μL of RNase-free water. PCR amplification was carried out at 95 °C for 5 min (initial denaturation), followed by 40 cycles at 95 °C for 30 s (denaturation), 58 °C for 20 s (annealing), and 72 °C for 20 s (fluorescence collection). To standardize gene expression, *β-actin* was utilized as the internal control, and relative gene expression levels were calculated using the 2^−∆∆Ct^ method. The data are recorded as mean ± standard error (SE) (*n =* 3).

### 2.6. Statistical Analysis

Statistical analysis of the results was conducted using SPSS Statistics 18.0 software (SPSS Inc., Chicago, IL, USA). Due to the small sample size (*n* = 13) in this study, the Shapiro–Wilk (S-W) test results were employed to assess normality (the threshold for conformity to a normal distribution is *p* > 0.05). For traits that conformed to a normal distribution, an independent *t*-test was used to compare differences between the FGs and SGs. Conversely, for traits that failed to meet the normality assumption (*p* < 0.05), the Mann–Whitney U test was conducted using the “Non-parametric Tests” module in SPSS; significant differences were considered to exist when *p* < 0.05.

## 3. Results

### 3.1. Analysis of Growth Data Between Fast-Growing (FG) and Slow-Growing (SG) Groups

**Figure 1:**
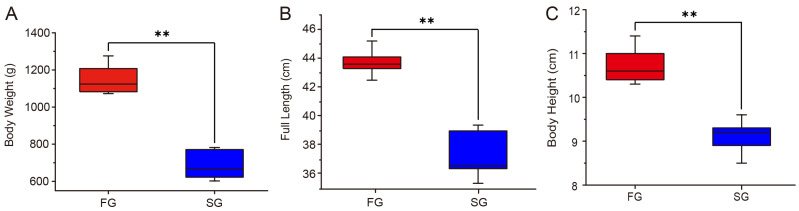
The statistics of body weight (**A**), full length (**B**), and body height (**C**) between the FG and SG. ** indicate statistical differences at *p* < 0.01. FG represents the fast-growing group (*n* = 7), and SG represents the slow-growing group (*n* = 6).

The body weight, full length, and body height of the fast-growing (FG) and slow-growing (SG) groups are shown in Figure 1. Specifically, the body weight (FG: 1149.1614 ± 28.13 g; SG: 686.76 ± 30.52 g), full length (FG: 43.74 ± 0.32 cm; SG: 37.32 ± 0.64 cm), and body height (FG: 10.70 ±0.38 cm; SG: 9.11 ± 0.15 cm) of the FG were significantly greater than those of the SG (*p* < 0.01) (Figure 1, Table 1).

### 3.2. Transcriptome Sequencing and Assembly

**Figure 2:**
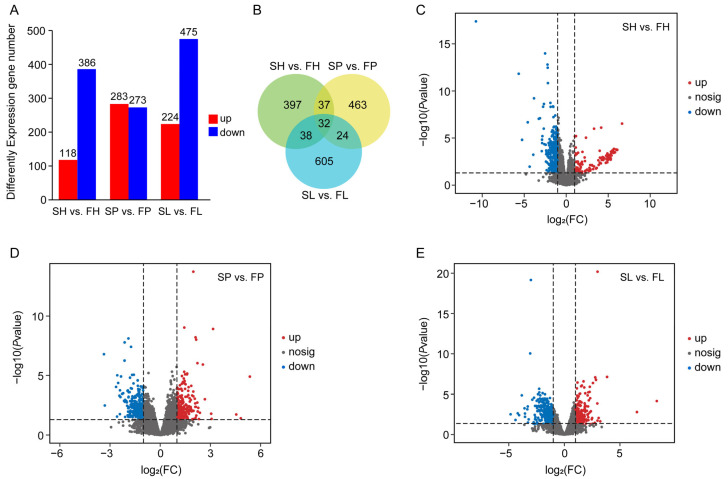
Gene expression in the hypothalamus, pituitary, and liver of greater amberjack with different growth rates. (**A**) Numbers of DEGs between SH vs. FH, SP vs. FP and SL vs. FL. Here SH and FH were the abbreviations of hypothalamus libraries from the SGs and FGs, SP and FP were the abbreviations of pituitary libraries from the SGs and FGs, and SL and FL were the abbreviations of liver libraries from the SGs and FGs. Red and blue bar charts represent up-regulated and down-regulated DEGs. (**B**) Venn diagram describes overlapping DEGs among hypothalamus, pituitary, and liver tissues from greater amberjack between the SGs and FGs. (**C**–**E**) is volcano plot, the red dots represent the up-regulated DEGs, the blue dots represent the down-regulated DEGs, the gray dots represent the no-regulated DEGs. The abscissa represents the logarithm of the difference coefficient, and the ordinate represents the logarithm of the *p*-value.

We performed a comparative transcriptome analysis of hypothalamus, pituitary, and liver tissues from 12-month-old greater amberjack in the FGs and SGs. An overview of the sequencing and assembly process is presented in Table 1. After removing low-quality or contaminated reads from the raw data, we obtained 90.25 Gb of clean reads. For the FG, we acquired 64.60 million reads for the H group, 62.43 million reads for the P group, and 68.01 million reads for the L groups, respectively. For the SG, we obtained 66.16 million reads for the H group, 69.62 million reads for the P group, and 72.36 million reads for the L group (Table 2). All samples exhibited Q20 frequencies of bases exceeding 97% and Q30 frequencies greater than 92%, as well as standard GC contents ranging from 46.65% to 50.11% (Table 2), indicating that the data used for subsequent analysis were of high quality. Overall, 772.95 million reads from the 18 sequencing libraries were successfully mapped to the assembled greater amberjack reference sequences, with approximately 88.64–92.23% of reads uniquely mapped to the greater amberjack genome (Table 2). PCA revealed that all biological replicates from each group clustered together, with 47.7%, 21.2%, and 4% of the variations explained by the first, second, and third principal components (PCs), respectively (Figure S1). We identified high-confidence DEGs in the hypothalamus, pituitary, and liver tissues of greater amberjack with varying growth rates, using a standard of |log_2_FC| > 1 and *p* < 0.05. The DEGs analysis revealed 504 DEGs between the FH and SH groups, comprising 118 DEGs up-regulated and 386 down-regulated genes (Figure 2A,C). Furthermore, 556 (283 up-regulated and 273 down-regulated) and 699 (224 up-regulated and 475 down-regulated) DEGs were identified between the FP and SP groups (Figure 2A,D), and the FL and SL groups, respectively (Figure 2A,E). A tissue-specific pattern was observed for the majority of DEGs across the three tissues. Notably, 32 DEGs were found to be shared between the SGs and FGs (Figure 2B). The top 10 up- and down-regulated DEGs for each phenotype group are provided in Table S2.

### 3.3. GO and KEGG Enrichment Analysis of DEGs

**Figure 3:**
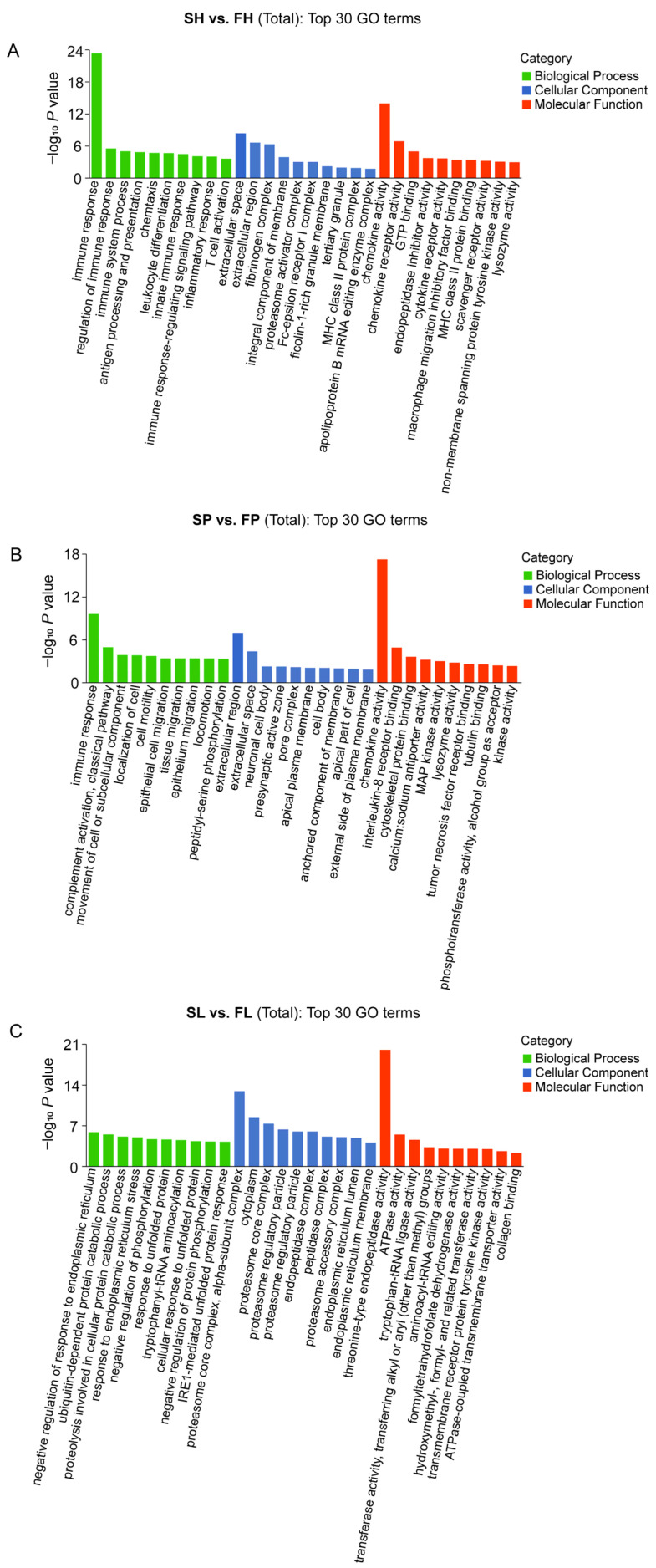
Gene ontology (GO) enrichment of DEGs in size-specific fish. (**A**) SH vs. FH; (**B**) SP vs. FP; (**C**) SL vs. FL. The *x*-axis represents the term name, and the *y*-axis represents the -log_10_ *p* value.

All the DEGs were annotated in the KEGG pathways and GO terms. The GO annotation was utilized to classify the DEGs into three categories: biological processes (BPs), cellular components (CCs), and molecular functions (MFs). A total of 347, 478, and 545 GO terms were significantly enriched in the hypothalamus, pituitary, and liver tissues of the greater amberjack between the fast- and slow-growth groups, respectively. Figure 3 and Tables S3–S5 illustrate the top 30 GO classifications of the DEGs in the three categories. The results of the GO analysis revealed significant alterations in immune response (GO:0006955) (BP), extracellular space (GO:0005615) and extracellular region (GO:0005576) (CC), and chemokine activity (GO:0008009) (MF) in the hypothalamus and pituitary tissues. Notably, the immune-related pathways were highly enriched, suggesting that the immune system may influence fish size. Furthermore, significant changes were observed in the negative regulation of response to endoplasmic reticulum stress (GO:1903573) (BP), proteasome core complex, alpha-subunit complex (GO:0019773) (CC), and threonine-type endopeptidase activity (GO:0004298) (MF) in the liver tissue.

**Figure 4:**
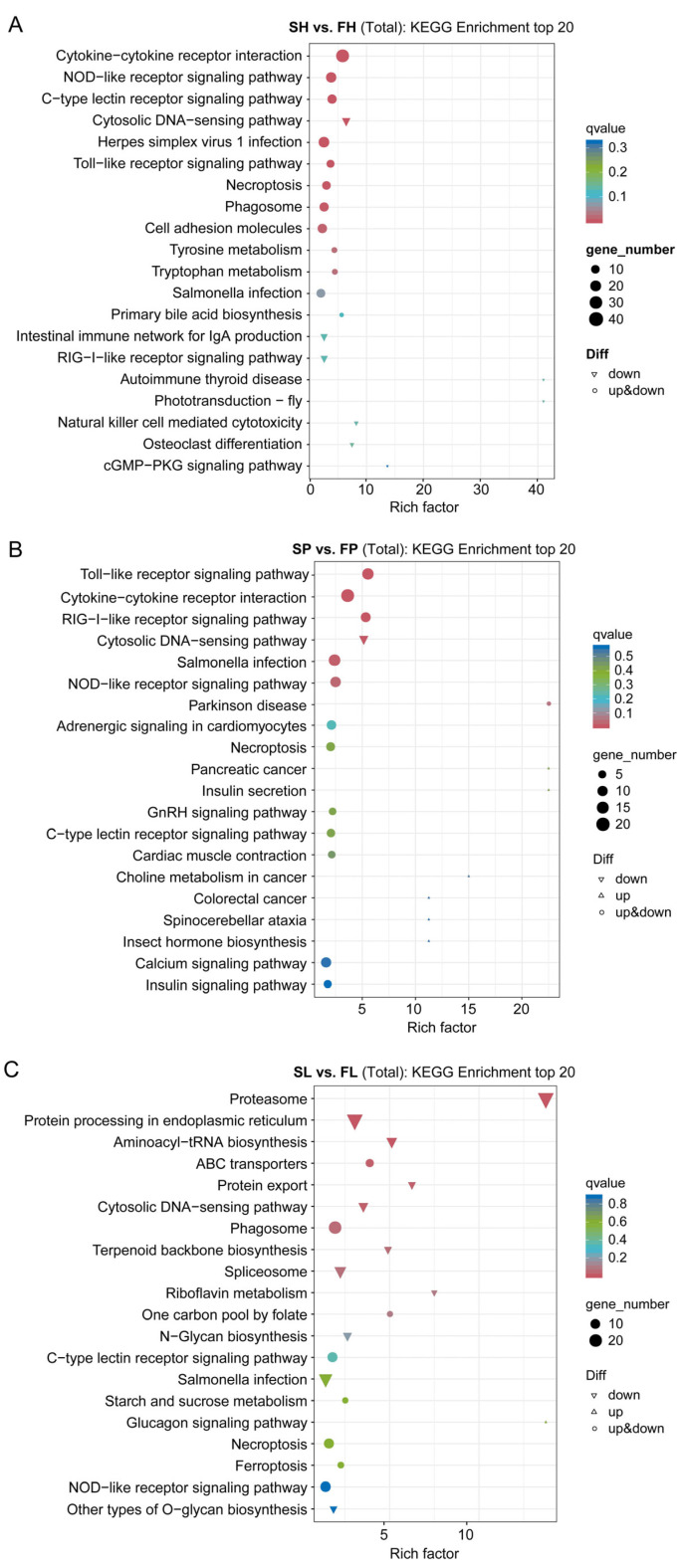
Kyoto Encyclopedia of Genes and Genomes (KEGG) pathway enrichment on DEGs-Bubble chart. (**A**) SH vs. FH; (**B**) SP vs. FP; (**C**) SL vs. FL. The *x*-axis represents the enrichment score. The *Y*-axis represents the KEGG pathway terms. The size of the dot represents the number of DEGs annotated in the pathway, and the color of the dot represents the *p* value of hypergeometric test.

The KEGG enrichment analysis was conducted to compare the DEGs between FH and SH, resulting in the identification of 114 enriched KEGG signaling pathways. Notably, 19 significant changes occurred in signaling molecules and interaction (cytokine–cytokine receptor interaction (ko04060) and cell adhesion molecules (ko04514)), immune system (NOD-like receptor signaling pathway (ko04621), C-type lectin receptor signaling pathway (ko04625), cytosolic DNA-sensing pathway (ko04623), and Toll-like receptor signaling pathway (ko04620), etc.), cell growth and death (necroptosis (ko04217)), and transport and catabolism (phagosome (ko04145)), and metabolism pathways (tryptophan metabolism (ko00380), tyrosine metabolism (ko00350), and primary bile acid biosynthesis (ko00120)), etc. (Figure 4A, Table S6). The number of DEGs in the signal transduction pathway was especially high. Furthermore, the KEGG enrichment analysis of the FP and SP DEGs yielded 128 enriched KEGG signaling pathways, with 13 significant changes noted in the immune system (Toll-like receptor signaling pathway (ko04620), RIG-I-like receptor signaling pathway (ko04622), Cytosolic DNA-sensing pathway (ko04623), and NOD-like receptor signaling pathway (ko04621)), signaling molecules and interaction (cytokine–cytokine receptor interaction (ko04060)), circulatory system (adrenergic signaling in cardiomyocytes (ko04261)), and cell growth and death (necroptosis (ko04217)), endocrine system (GnRH signaling pathway (ko04912) and insulin secretion (ko04911)), etc. (Figure 4B, Table S6). In FL vs. SL, the KEGG enrichment analysis generated 148 KEGG signaling pathways, with 13 significant changes occurred in folding, sorting, and degradation (proteasome (ko03050), protein processing in endoplasmic reticulum (ko04141), and protein export (ko03060), translation (aminoacyl-tRNA biosynthesis (ko00970)), immune system (cytosolic DNA-sensing pathway (ko046230, C-type lectin receptor signaling pathway (ko04625)), transport and catabolism (phagosome (ko04145)), and metabolism (terpenoid backbone biosynthesis (ko00900), riboflavin metabolism (ko00740), one carbon pool by folate (ko00670), and N-Glycan biosynthesis (ko00510)), etc. (Figure 4C, Table S6). Additionally, the top 20 enriched signaling pathways were analyzed, highlighting “Cytokine-cytokine receptor interaction”, “Toll-like receptor signaling pathway”, and “Proteasome” as the most prominent pathways in SH vs. FH, SP vs. FP, and SL vs. FL, respectively (Figure 4, Table S6).

### 3.4. qPCR of Candidate GH/IGF Axis Related Genes

**Figure 5:**
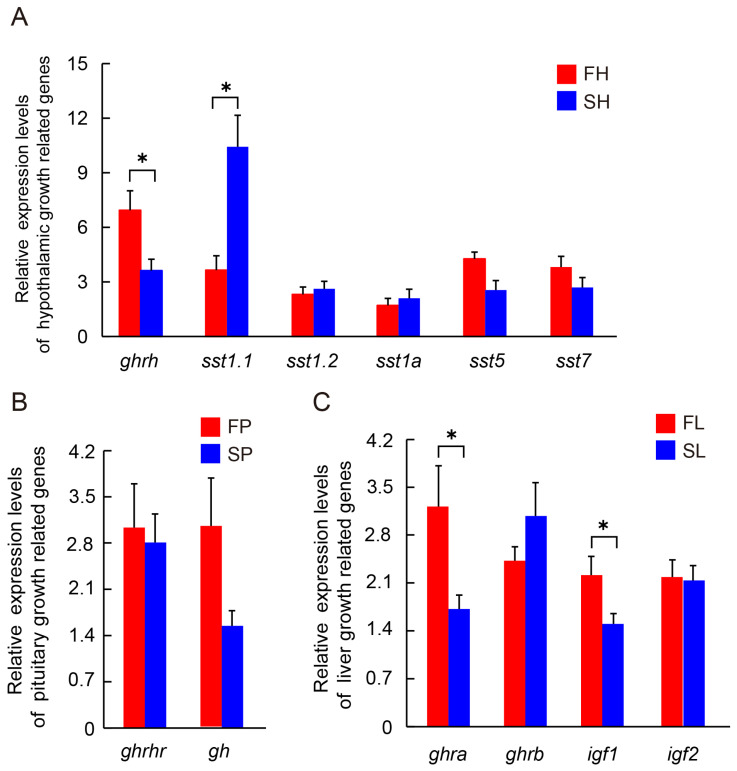
Expression patterns of hypothalamus (**A**), pituitary (**B**), and liver (**C**) axis growth-related genes in growth-differentiated individuals of greater amberjack. Gene expression level was presented as an average normalized ratio (*n* = 5, ±SE). *β-actin* serves as the reference gene. * indicate statistical differences at *p* < 0.05, as determined by Independent-Samples *t*-Test.

In the hypothalamus, decreased levels of *ghrh* and increased expression of *sst1.1* mRNA were observed in the SG compared to the FG. However, no significant differences were observed in the expression levels of *sst1.2*, *sst1a*, *sst5*, and *sst7* mRNA between the two groups (Figure 5A). In the pituitary gland, the expression levels of *gh* and *ghrhr* mRNA showed no significant differences between the two groups (Figure 5B). In the liver, the expression of *ghrα* and *igf1* mRNA was significantly reduced in the SG compared to the FG. Furthermore, there was no significant difference in the expression levels of *igf2* and *ghrb* mRNA between the two groups (Figure 5C).

### 3.5. Validation by qPCR

**Figure 6:**
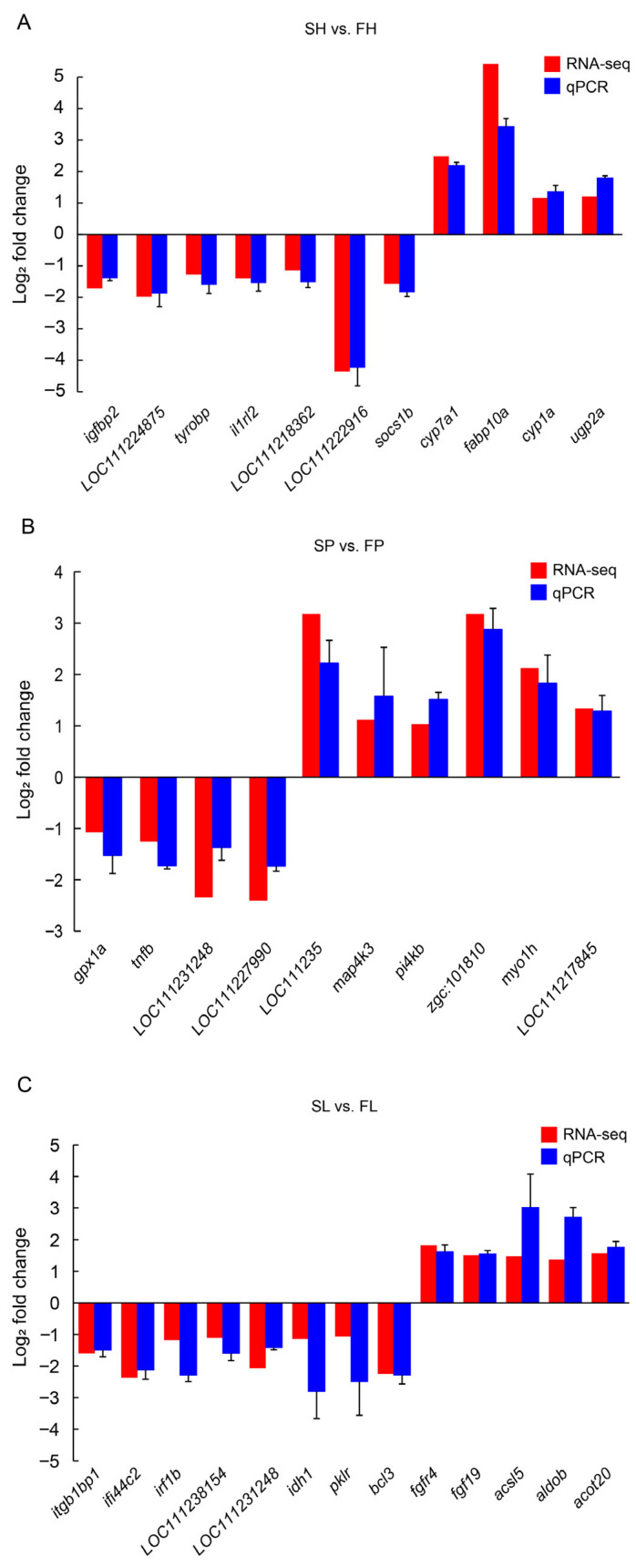
Comparison of gene expression patterns obtained using RNA-seq analysis and qRT-PCR. (**A**) qPCR of DEGs from hypothalamus between SGs and FGs. (**B**) qPCR of DEGs from pituitary between SGs and FGs. (**C**) qPCR of DEGs from liver between SGs and FGs. Gene expression level was presented as an average normalized ratio (*n* = 3, ±SE). *β-actin* serves as the reference gene.

qPCR was performed to assess and validate the expression levels of the 33 DEGs identified through RNA-Seq, alongside one endogenous reference gene. The fold changes obtained from qPCR were compared to those derived from RNA-Seq. The expression trends of the DEGs identified by qPCR closely mirrored those determined by RNA-Seq (Figure 6). The collective data (combined for the hypothalamus, pituitary, and liver) are presented in the scatterplot in Figure S2, which shows a positive correlation between the expression values calculated by each analysis (*p* < 0.001, R^2^ = 0.874) (Figure S2). Therefore, RNA-seq is a reliable method for analyzing the differential expression of mRNA.

**Figure 7:**
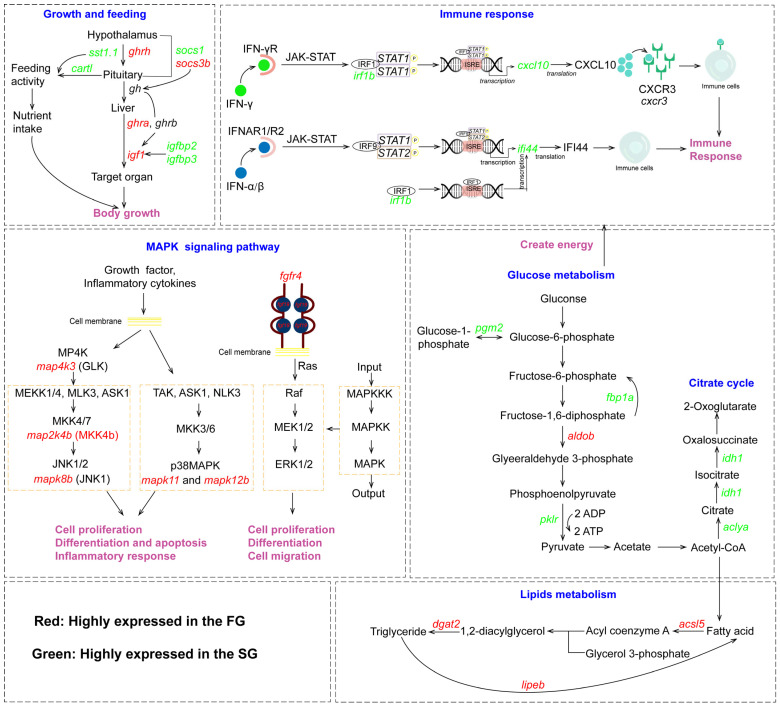
Schematic representation of DEGs in growth-related pathways. Red and green colors indicate the up- and down-regulation of DEGs in the FG, respectively.

A thorough analysis of the transcriptome provided a comprehensive understanding of the genes involved in the growth of greater amberjack (Figure 7). The results showed that pathways related to growth, metabolism, and immunity were enriched. Genes associated with growth (*ghrh*, *ghra*, *igf1*), cell proliferation (*fgf19*, *fgfr4*, *mapk8b*, *map2k4b*, and *map4k3*), and lipid metabolism (*acsl5*, *dgat2*, *lipeb*, *cyp7a1*, and *fabp10a*) were up-regulated in the FG compared to the SG. Conversely, glycolysis (*fbp1a*, *pklr*, *pgm2*), citrate cycle (TCA cycle) (*aclya*, *idh1*), and immune-related genes (*irf1b*, *cxcl10*, *ifi44*, *mapk11*, and *mapk12b*) were up-regulated in the SG compared to the FG, which can activate the immune system and hinder growth during an immune response.

## 4. Discussion

The growth rate of aquatic animals is regulated by numerous genes and influenced by complex environmental factors. Growth is a significant economic trait in aquaculture and has been prioritized in the development of selective breeding programs [11]. Consequently, elucidating the underlying mechanisms of its regulation from both genetic and economic perspectives is particularly important [24,25]. The brain (including the hypothalamus and pituitary gland) is the most advanced part of the nervous system and regulates various life activities in fish, such as growth, development, digestion, and immunity [26,27]. The liver is a vital organ that plays a crucial role in metabolism and immune function [28]. Variations in fish growth rates may be reflected in the RNA profiles of brain and liver tissues. Therefore, exploring the DEGs in the brains and livers of FG and SG greater amberjack can help identify the mechanisms that significantly influence their growth rates.

### 4.1. Expression of Growth-Related Genes

The growth and development of fish involve the organic regulation of myocyte proliferation, differentiation, and apoptosis, primarily governed by the GH/IGF system [29]. This system is known to positively regulate growth in vertebrates. It is well established that GHRH in the hypothalamus stimulates GH production in the pituitary, which binds to liver GHR, and increases the production and release of IGF-I from the liver into the systemic circulation of both mammals and fish [30]. IGF-1 is closely associated with growth performance in teleosts, and its up-regulation can promote cell proliferation, differentiation, and protein synthesis by activating downstream PI3K-Akt and MAPK signaling pathways, thereby accelerating somatic growth [31,32]. In our study, the up-regulated expression of *ghrh* and *igf1* in the FG suggests that the somatotropic axis plays a pivotal role in driving growth divergence in greater amberjack, similar to findings in channel catfish [33] and rainbow trout (*Oncorhynchus mykiss*) [34]. In humans and salmonids, approximately 99% of circulating IGF-1 is bound to IGF-binding proteins (IGFBPs) [35]. These binding proteins extend the half-life of IGF-1 in serum, facilitate or inhibit its binding to receptors in peripheral tissues, and assist in its sequestration across various tissue types [36]. Both IGFBP-2 and -3 can inhibit or potentiate IGF actions. In adult zebrafish, IGFBP-2 significantly inhibited IGF-I-stimulated cell proliferation and DNA synthesis, while total serum IGF-1 levels were reduced in rainbow trout IGFBP-2b knockout lines [36,37]. IGFBP-3 can enhance or inhibit IGF-1 signaling depending on the tissue context and post-translational modifications [37,38,39]. The RNA-seq results indicated down-regulation of *igfbp2* and *igfbp3* mRNA in the FG, suggesting that these factors may act as negative growth regulators downstream in the GH-IGF-1 axis in greater amberjack. GHRs are transmembrane receptors for growth hormone that play a crucial role in regulating body growth and metabolism. In species such as rainbow trout [40], channel catfish [12], grass carp (*Ctenopharyngodon idella*) [41], and largemouth bass (*Micropterus salmoides*) [42], down-regulation of *ghrb* was observed in the FG, suggesting that *ghrb* may serve as a negative indicator for rapid growth in fish. It is worth noting that the two types of GH receptors are regulated differently in greater amberjack; *ghrα* was up-regulated, while *ghrb* exhibited no significant difference between the FL and SL groups. These findings imply that there is unique functionality associated with the GHR subtypes, with *ghrα* appearing to play a positive role in promoting rapid growth in greater amberjack.

The growth of fish is influenced by the metabolism and transformation of various nutrients within their bodies, with feeding being a crucial method through which fish acquire these nutrients [43]. Cocaine- and amphetamine-regulated transcript (CART) is a potent anorectic peptide found in both mammals and fish [44,45]. Thus, the activation of *cartl* in the SG may inhibit the feeding activity of greater amberjack, leading to insufficient nutrient intake, and consequently slower growth and development, which is consistent with findings in Chinese longsnout catfish [13]. Somatostatin (SST) inhibits body growth by suppressing the GH-IGF-1 axis and affects glycolipid metabolism by reducing insulin and glucagon secretion [8,46]. Furthermore, SST regulates feeding behaviors in fish by inhibiting the release of specific gastrointestinal peptides [46]. In this study, *sst1.1* mRNA was highly expressed in the SG, indicating that it may influence the growth of greater amberjack by inhibiting GH secretion and feeding activity.

### 4.2. Molecular Signaling Pathways for Growth

In this study, several prominent pathways, including the mitogen-activated protein kinase (MAPK) and insulin signaling pathways, were shown to be linked with growth regulation and development. The MAPK signal transduction pathway is a ubiquitous and highly conserved mechanism that regulates eukaryotic cell development. c-Jun N-terminal protein kinase (JNK), a subfamily of the MAPK super-family, is activated by sequential protein phosphorylation through a MAP kinase module: MAP3K → MAP2K → MAPK [47,48]. The JNK signaling pathway plays a significant role in various cellular processes, including cell proliferation, differentiation, and apoptosis [47,48]. Moreover, as upstream regulators of the MAP kinase cascade, GLK and other MAP4Ks activate JNK in response to environmental stress and pro-inflammatory cytokines in cultured cell lines [49,50]. In greater amberjack, the expression of *mapk8b* (JNK1), *map2k4b* (MKK4b), and *map4k3* (GLK) was up-regulated in the pituitary of the FG, indicating that the JNK signaling pathway may play a crucial role in the growth of greater amberjack by regulating cell proliferation and apoptosis. Furthermore, transcriptomic analyses revealed that the fibroblast growth factor 19 (*fgf19*) and FGF receptor 4 (*fgfr4*) genes in the MAPK pathway were up-regulated in the liver of larger fish. FGFs are secretory cytokines with diverse biological activities. FGF19, a high-affinity, heparin-dependent ligand for FGFR4, plays an essential role in various biological activities, including cell proliferation, differentiation, growth, and morphogenesis, as well as the regulation of lipid metabolism [51,52]. The expression levels of *fgfr4* and *fgf19* were up-regulated in the livers of the FG, suggesting that *fgf19* acts through *fgfr4* to promote cell proliferation and contribute to fish metabolism via the MAPK pathway in fast-growing greater amberjack.

The insulin/insulin-like growth factor signaling pathway (IIS) is highly conserved across vertebrates and invertebrates, regulating multiple physiological processes, including metabolism, growth, and development [53]. The suppressors of cytokine signaling (SOCS) family of proteins serves as crucial negative regulators of cytokine and growth factor signaling pathways [54]. Members of this family establish a classical negative feedback loop, primarily involving the inhibition of the insulin signaling pathway and the JAK-STAT signaling cascade [55,56]. Extensive analyses have implicated both SOCS1 and SOCS3 as significant regulators of GH signal transduction. These SOCS proteins are induced in cells stimulated by GH and their over-expression in cell lines obstructs aspects of GH signaling [13,55]. In the FG, the *socs1* mRNA was down-regulated in both the hypothalamus and pituitary, while the *socs3b* mRNA was up-regulated in the pituitary. This suggests that these two genes may play an essential role in the growth and development of greater amberjack by regulating the GH signaling pathway.

### 4.3. DEGs Related to Lipids Metabolism

Lipids are high-energy compounds that play a crucial role in the growth and development of fish, as well as in various processes related to digestion, physiology, and metabolism [57]. In this study, several genes associated with lipid synthesis were identified, including acyl-CoA synthetase long-chain family member 5 (*acsl5*) and diacylglycerol acyltransferase 2 (*dgat2*) and lipase, hormone-sensitive b (*lipe*). ACSL5 is a significant regulatory factor that mediates the fatty acid channels between lipid synthesis and the β-oxidation pathway [58]. The overexpression of ACSL5 in hepatoma cells primarily enhances the conversion of fatty acids to triacylglycerol (TG), while the knockdown of ACSL5 in isolated rat hepatocytes leads to reduced TG accumulation and increased fat oxidation [59]. DGAT2 catalyzes the final step of TG synthesis. In mice, the inhibition of DGAT2 has been shown to decrease fatty acid synthesis and reduce TG accumulation and secretion from the liver [60]. Lipe is a critical metabolic enzyme and an important regulator of lipolysis in adipocytes. It catalyzes the release of fatty acids from stored triglycerides in adipocytes, thereby facilitating fat metabolism [61]. In our study, the elevated expression levels of *acsl5*, *dgat2*, and *lipeb* in the FG indicate that these genes may promote FG growth by stimulating lipid synthesis and accelerating lipid breakdown to provide energy. Furthermore, genes related to cholesterol metabolism, such as squalene epoxidase (*sqle*) and cholesterol 7alpha-hydroxylase (*cyp7a1*), exhibited significant changes. SQLE, located in the endoplasmic reticulum, is responsible for the initial oxygenation step in sterol biosynthesis and is considered one of the key enzymes in this pathway, significantly influencing the flux of cholesterol synthesis [62]. CYP7A1 is the rate limiting enzyme in the bile acid biosynthetic pathway, converting cholesterol into bile acids, which represent a major output pathway for cholesterol catabolism [63]. In the FG, the high expression of *cyp7a1* in the pituitary and the low expression of *sqle* in the liver indicate an increase in cholesterol catabolism, which is beneficial for promoting lipid digestion and absorption. Fatty acid binding proteins (FABPs) play an important role in regulating the transport and metabolism of lipid-like molecules [64]. In orange-spotted grouper (*Epinephelus coioides*), an additive diet containing FABP10 can modulate lipid metabolism and contribute to ATP supply in cold environments [65]. This study found that *fabp10a* was significantly up-regulated in the FG, suggesting that the rapid growth of greater amberjack may requires more active lipid metabolism.

### 4.4. DEGs Related to Immune Response

Commercially reared animals encounter serial immune challenges throughout their growth. Due to the diversion of nutrients away from growth to support immune-related functions, these immune challenges are significant barriers to achieving the animals’ genetic potential for growth and efficiency of gain [66]. Consequently, the reduced growth observed in the SG may partially result from the considerable energy consumption required to initiate immune responses to environmental stressors. In the SG, genes associated with the immune response were expressed, with numerous genes exhibiting significant activation and up-regulation, such as C-X-C motif chemokine-like 10 (*cxcl10*), interferon (IFN) regulatory factor 1b (*irf1b*) and interferon-induced protein 44 (*ifi44*). CXCL10 (also known as interferon-γ-inducible protein 10), IRF1, and IFI44 are involved in the regulation of innate and adaptive immune responses [67,68,69]. CXCL10 was initially identified as a pro-inflammatory chemokine, functioning primarily as a “chemical beacon” or “recruitment signal”. By binding to its receptor CXCR3, it recruits specific immune cells—particularly T cells and NK cells—to sites of inflammation or infection, thereby playing a pivotal role in immune responses [68]. IRF-1 is a master transcription factor in the interferon-γ pathway that regulates both innate and adaptive immune responses [67]. In murine models, IRF-1 mRNA expression positively correlates with CXCL10 and CXCR3 in hepatocellular carcinoma (HCC) tumors, where HCC-derived IRF-1 activates immune cells to induce apoptosis of tumor cells through the CXCL10/CXCR3 axis [70]. In the greater amberjack, *cxcl10* and *irf1b* are highly expressed in the hypothalamus and liver of the SG, facilitating the production of immune cells to address environmental stress by activating the CXCL10/CXCR3 axis. IFI44 is a member of the type I IFN-inducible gene family, whose expression is induced by IFN-α/β (key regulators of immune activation) and in turn mediates the type I IFN-inducible gene pathway [69,71]. IFN-I can protect the host against some bacterial infections. Additionally, IRF1 can directly bind to the IFN-stimulated response element in the IFI44L promoter, thereby activating IFI44 [72]. Lysozyme (LYS), a nonspecific immune factor with potent antibacterial properties [73], kills bacteria by hydrolyzing the peptidoglycan layer of their cell walls, playing a vital role in innate immunity [74]. Both *ifi44* and *lysg* are highly expressed in the hypothalamus, pituitary, and liver of the SG, indicating their crucial role in resisting bacterial and viral invasion.

Tumor necrosis factor (TNF), a family of cellular signaling proteins involved in systemic inflammation, is one of the most critical pro-inflammatory cytokines, which is necessary for efficient innate and adaptive immune responses [75]. TNF-β, also known as lymphotoxin-alpha (LT-α), is primarily produced by activated T and B lymphocytes [76]. TNF-β participates in the adaptive immune response of Nile tilapia (*Oreochromis niloticus*) lymphocyte by initiating cell apoptosis [75]. The p38 kinases are one of the four subgroups of the MAPK super-family and play a crucial role in innate immunity. Evidence indicates that the p38b MAPK pathway regulates the expression of several pro-inflammatory cytokines, including TNF-α and IL-1β, through the phosphorylate on various transcription factors and translational regulators [77,78]. This study reveals that *tnfb*, *mapk11*, and *mapk12b* (both p38 MAPK isoforms) were significantly up-regulated in the SG, thereby enhancing the immune response of the pituitary. Following pathogen invasion, immune cells rapidly respond to antigens through swift proliferation and functional execution, which requires nutrients to sustain the underlying cellular pathways, rendering the immune response a highly energy-intensive process [79]. Concurrent with immune activation, we observed up-regulated expression of glycolysis (*fbp1a*, *pklr*, *pgm2*) and citrate cycle (TCA cycle)-related genes (*aclya*, *idh1*) in smaller individuals, indicating the activation of energy metabolism pathways. These findings suggest that once the immune system is activated, nutrients and energy typically allocated for growth may be redirected towards the production of immune responses. The up-regulation of immune-related genes indicates elevated stress levels in the SG.

In summary, our study provides a comprehensive transcriptional profile associated with divergent growth phenotypes in sub-adult greater amberjack. These differences likely encompass both sustained regulatory features established during early development and adaptive responses in the current growth state. Fundamental growth-regulatory pathways, such as the GH/IGF axis and insulin/insulin-like growth factor signaling pathways are activated during early embryogenesis and larval development in fish, which can produce long-term growth potential [80,81]. The sustained differential expression of key regulators (e.g., *ghrh*, *ghra*, *igf1*, *igfbp2*, and *igfbp3*) in 12-month-old fish may therefore reflect a maintained molecular signature of developmental programming that originated earlier. Concurrently, genes involved in immediate processes such as lipid and cellular energy metabolism, and immune responses showed differential expression between the two groups. These processes are highly correlated with growth rate and are typically considered hallmarks of the current anabolic or catabolic state [8,10,13]. These factors may be more consequential for maintaining, rather than initiating, growth differentials. While our research limits the ability to fully trace the early initiation of growth differences, the current results lay a solid foundation for further exploration of the temporal dynamics of growth-related gene regulation.

## 5. Conclusions

In conclusion, we conducted comparative transcriptome analyses to explore the functions of the hypothalamus–pituitary–liver axis in growth regulation in greater amberjack. A total of 504, 556, and 699 DEGs were identified in the hypothalamus, pituitary, and liver between the FGs and SGs, respectively. Functional analysis indicated that the FG exhibited an active lipid metabolism capacity, while the SG showed consumption of additional energy in response to environmental stress, with hindered growth due to the generation of immune responses. Furthermore, the FG demonstrated stronger cell proliferation abilities and feeding behavior than the SG. The results of this study enhance our understanding of the genetic mechanisms and regulatory pathways underlying differential growth rates and also provide candidate genes as potential biomarkers for growth in selective breeding programs of greater amberjack.

## Figures and Tables

**Table 1:** Phenotypic data of the greater amberjack in the fast-growing group (FG) and slow-growing group (SG).

Groups	Full Length (cm)	Body Height (cm)	Body Weight (g)
Fast growing group/FG			
F1 *	43.4	10.3	1207.34
F2 *	42.5	10.6	1072.31
F3 *	43.6	10.4	1123.84
F4	44.1	10.6	1104.7
F5	45.2	11	1275.9
F6	43.3	10.6	1082.41
F7	44.1	11.4	1177.63
Slow growing group/SG			
S1 *	36.6	9.6	622.78
S2 *	35.4	8.9	602.74
S3 *	39.4	9.3	782.31
S4	37.1	8.5	673.4
S5	36.4	9.2	668.1
S6	39.0	9.2	771.24

Note: Individuals marked with * represent transcriptome sequencing and qPCR validation experiments samples.

**Table 2:** Statistics of sequencing data.

Sample Name ^a^	Clean Reads (10^6^)	Clean Base (10^6^)	Q20 (%) ^b^	Q30 (%) ^c^	GC Contents (%)	Total Mapped (10^6^) ^d^	Uniquely Mapped (10^6^) ^e^
FH-1	22.20	66.15	98.21	95.06	46.67	42.56 (95.84%)	40.00 (90.09%)
FH-2	21.51	63.98	98.39	95.44	47.25	41.33 (96.08%)	38.13 (88.64%)
FH-3	20.89	62.17	98.38	95.46	46.91	40.17 (96.15%)	37.46 (89.68%)
SH-1	24.73	73.61	98.35	95.28	46.65	47.38 (95.80%)	44.12 (89.20%)
SH-2	22.10	65.79	98.15	94.90	46.81	42.17 (95.39%)	39.38 (89.09%)
SH-3	19.33	57.53	98.37	95.40	47.16	37.01 (95.75%)	34.60 (89.49%)
FP-1	20.73	61.59	98.67	96.18	48.40	39.88 (96.20%)	38.17 (92.08%)
FP-2	21.28	63.29	98.54	95.80	48.24	41.02 (96.39%)	39.11 (91.90%)
FP-3	20.42	60.78	98.5	95.63	48.15	39.07 (95.66%)	37.67 (92.23%)
SP-1	19.57	58.28	98.5	95.64	48.49	37.43 (95.63%)	35.70 (91.19%)
SP-2	22.93	68.36	98.36	95.39	47.00	44.09 (96.14%)	42.07 (91.72%)
SP-3	27.12	80.75	97.14	92.00	47.56	51.23 (94.45%)	49.18 (90.67%)
FL-1	22.79	67.89	98.41	95.54	49.38	43.74 (95.94%)	40.66 (89.19%)
FL-2	21.29	63.39	98.5	95.69	50.11	40.84 (95.90%)	37.95 (89.11%)
FL-3	23.93	71.29	98.38	95.46	48.56	45.95 (96.03%)	42.59 (89.01%)
SL-1	21.41	63.73	98.55	95.72	48.69	41.30 (96.45%)	44.12 (89.20%)
SL-2	24.20	72.23	98.27	95.09	49.31	46.53 (96.12%)	39.38 (89.09%)
SL-3	26.75	79.81	97.39	92.72	49.17	51.24 (95.79%)	42.76 (90.13%)

^a^ 1, 2, and 3: Three independent biological replicates. ^b^ Q20: The percentage of bases with a Phred value > 20. ^c^ Q30: The percentage of bases with a Phred value > 30. ^d^ The number of clean reads that mapped onto the annotated genome. ^e^ The number of clean reads that uniquely mapped onto the annotated genome.

## Data Availability

The data that support the findings of this study are available upon reasonable request. The raw reads used in this article have been deposited into the Sequence Read Archive (SRA) of the NCBI database under BioProject accession number: PRJNA1320817: SRR35276430–SRR35276447).

## Supplementary Materials

The following supporting information can be downloaded at: https://www.mdpi.com/article/10.3390/ani16030516/s1. Figure S1. Principal component analysis (PCA) showing the differences between different biological groups. Figure S2. Scatterplot of relative transcript abundance (log_2_(fold change)) calculated by RNA-seq versus RT-PCR analysis. Table S1. The primers used in this study for qPCR. Table S2. List of the top 10 successfully annotated up- and down-regulated DEGs in the hypothalamus, pituitary, and liver tissues of Fast- (F) and Slow- (S) growing greater amberjack. Table S3. GO analysis of DEGs in the SH vs. FH comparison group in this study. Table S4. GO analysis of DEGs in the SP vs. FP comparison group in this study. Table S5. GO analysis of DEGs in the SL vs. FL comparison group in this study. Table S6. DEGs of significantly enriched KEGG-Categories between fast- and slow-growing greater amberjack.
