# Supplementary material for: Multi-Organ Transcriptomic Analysis of Greater Amberjack (Seriola dumerili) with Different Growth Rates
Source: Animals (Basel). 2026 Feb 6;16(3):516. doi: 10.3390/ani16030516 (PMC12896609; doi:10.3390/ani16030516)
Supplement: Supplementary file 1 [file animals-16-00516-s001.zip › Supplementary figures.pdf]

Supplementary material

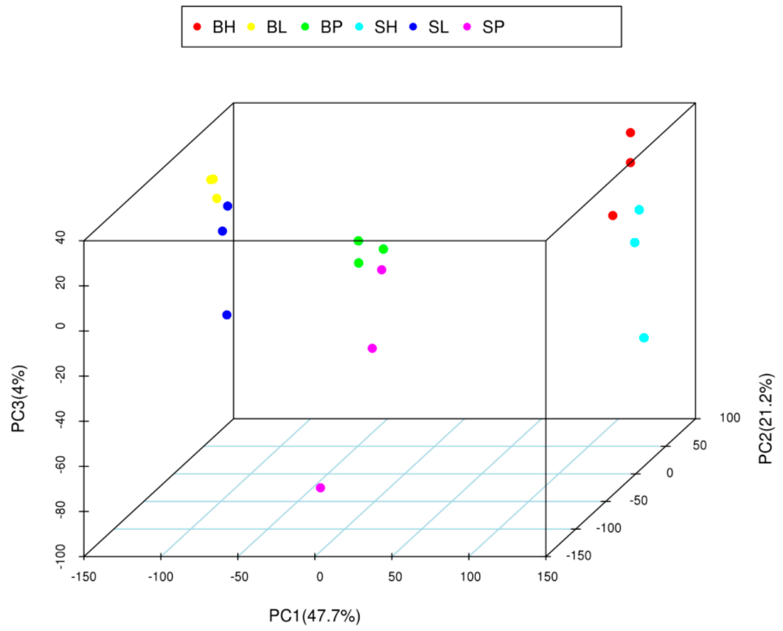

Figure. S1 Principal component analysis (PCA) showing the differences between different biological groups.

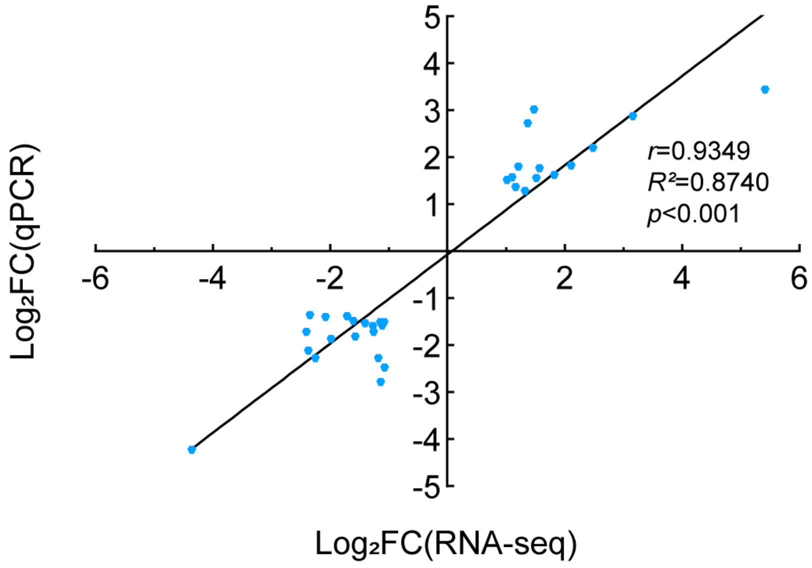

Figure. S2 Scatterplot of relative transcript abundance (log2(fold change)) calculated by RNA-seq versus RT-PCR analysis.
